# A Model School-Room

**Published:** 1876-07

**Authors:** 


### A MODEL SCHOOL-ROOM

It was a room about twenty feet square, surrounded on all sides by desks, at which were seated as many boys as could be comfortably ranged along the walls—say thirty in number. In the centre burned a coal stove, with furious heat; and just beside it was the platform, with the teacher's desk and apparatus on it. There was about enough vacant space allowed a class to stand up for recitation, very little more.

It was about the hour of closing school. I knocked at the door, and, as I put my foot over the threshold, the stifling air almost forced me back, as if I were entering a subterranean cavern. There were two windows in the room, closed fast against the intrusion of a breath of Heaven's pure air. The vapor stood in thick drops on every pane of glass. I did not see a ruddy-faced boy in the whole company. They were a sallow, sickly, cadaverous-looking set, fast making ready for the grave of consumptives.

What a pity! Thirty fine boys, as ever started on the journey of life, imprisoned for seven or eight hours a day in this foul vapor-bath—all to force their intellects, as horticulturists force plants, in the stifling air of a hot-house. Who were the parents of these boys, that they would permit them thus to be suffocated, body and mind? The wealthiest in the city, who spend fortunes for the education of their children, but will not invest a cent to provide for the poor little sufferers a gallon or two of fresh air.

